# Use of novel drug therapies during pregnancy and breast-feeding

**DOI:** 10.1016/j.crwh.2023.e00511

**Published:** 2023-05-03

**Authors:** Paul T.-Y. Ayuk

**Affiliations:** The Newcastle-upon-Tyne Hospitals NHS Trust, Newcastle NE1 4LP, UK

**Keywords:** Pregnancy, Breast-feeding, Novel medical therapies, Teratogenicity

According to the US Centers for Disease Control and Prevention (CDC), 9 out of 10 women in the US take medications during pregnancy, but fewer than 10% of medications have enough information to determine fetal risk [1]. The challenges with medication use during pregnancy extend beyond uncertainties about fetal risks. Pregnancy is associated with major physiological changes, including changes in blood and plasma volume, renal and cardiac function, and the concentrations of clotting factors and a variety of hormones, all of which may alter the pharmacokinetics and pharmacodynamics of drugs. Yet there are very few drugs with sufficient information to determine if the dose regimens for non-pregnant patients are appropriate for pregnant and post-partum women. These problems are particularly acute when the use of novel (usually safer and more cost-effective) drugs is being considered. Finally, initiatives such as Accelerating Innovations for Mothers [2] recognise the very limited progress in developing novel drug therapies for pregnancy-specific conditions such as pre-eclampsia, preterm labour and fetal growth restriction. Consequently, women and clinicians are often faced with difficult choices when considering the use of novel medical therapies during pregnancy and breast-feeding.

For very good reasons, pregnant and breast-feeding women are excluded from early-phase drug trials. What is of concern is the time interval between a new drug being licensed for use in non-pregnant patients and high-quality data on safety during pregnancy and breast-feeding becoming available. For example, direct oral anti-coagulants (DOACs) have been used as alternatives to heparin and warfarin for a variety of indications for nearly a quarter of a century [3]. Yet it is only in recent years that the safety and efficacy of DOACs for post-partum thromboprophylaxis have been evaluated [4]. Ondansetron, on the other hand, has been used to treat severe nausea and vomiting in pregnancy since the 1990s and has been presumed to be safe for the fetus. Yet it was not until 2018 that a signal of teratogenicity was detected [5]. Detecting adverse drug effects on cognitive function or the function of other critical organs and pathways is likely to be more challenging and take longer than detecting associations with structural abnormalities. This is the case with sodium valproate, where the association with a variety of fetal structural abnormalities has been recognised for a long time. However, the association between valproate exposure in-utero and neurodevelopmental delay / autism has only recently been recognised [6]. Impairments that are purely functional may have a greater impact on quality of life than anatomical malformations, which in many cases are amenable to surgical correction. When novel medical therapies are being evaluated for use in pregnancy, the absence of fetal structural abnormalities does not imply the drug is safe for the fetus.

Despite the challenges, there is a recognised need to develop novel therapies for pregnancy-specific conditions and give pregnant and breast-feeding women safe access to the best medical therapies. The first port of call for women and clinicians seeking information on medication use during pregnancy and breast-feeding is the prescription drug labelling (US Food and Drug Administration, FDA) or the Summary of Product Characteristics (SmPC) for the European Medicines Agency (EMA). A recent review of 31 medications found that human data on pregnancy and lactation were not available on any of the medications at the time of approval, with such data being added as studies were conducted post-approval [7]. Women and clinicians considering the use of novel medical therapies are unlikely to find the SmPC or similar documents useful. The earliest data on the use of novel medical therapies during pregnancy and breast-feeding are likely to come from case reports. There is a recognised bias in favour of clinicians submitting, and journals accepting, case reports with a successful pregnancy outcome. It is recommended that clinicians report, and journals publish, reports on the use of novel medical therapies during pregnancy even when pregnancy has been unsuccessful, and especially when there is a potential link between medication therapy and pregnancy outcome. This will ensure that the published literature gives a more balanced assessment of the consequences of therapy during pregnancy.

Analysis of data from drug registries and large population studies currently provide good-quality evidence for the safety of novel therapies during pregnancy and breast-feeding. The prescription drug labelling (FDA) but not the SmPC (EMA) [7] contains information on pregnancy exposure registries where available. Clinicians and women may therefore not be aware of the existence of relevant registries. Furthermore, it takes several years, maybe decades, before registries contain sufficient data for analysis. During this period, case reports and case series may be the only information available to women and clinicians.

Many women are understandably reluctant to take any medication during pregnancy and breast-feeding, and clinicians recognise the need to prescribe medication only when absolutely necessary. Nevertheless, large numbers of women take medications during pregnancy with potential benefits and risks to the woman, fetus and neonate. For many medications, a body of evidence exists that enables a balanced judgement on benefits and risks. For novel therapies, however, there are likely to be very limited human data on safety, and efficacy at the non-pregnancy dose cannot be assumed. When women who are using such novel therapies find themselves unexpectedly pregnant, or a judgement is made to initiate or continue such therapies during pregnancy, data should be added to drug registries where available and case reports submitted for publication. Current clinical trials to elucidate the pharmacokinetics of commonly used medications during pregnancy are timely and will enable prescribers to take account of the physiological changes during pregnancy. The use of physiologically-based pharmacokinetic-pharmacodynamic modelling might mean such clinical trials are unnecessary in future.
